# Statin Use and Benefits of Thyroid Function: A Retrospective Cohort Study

**DOI:** 10.3389/fendo.2021.578909

**Published:** 2021-03-02

**Authors:** Yupeng Wang, Qihang Li, Zhongshang Yuan, Shizhan Ma, Shanshan Shao, Yafei Wu, Zhixiang Wang, Qiu Li, Ling Gao, Meng Zhao, Jiajun Zhao

**Affiliations:** ^1^Department of Endocrinology, Shandong Provincial Hospital, Cheeloo College of Medicine, Shandong University, Jinan, China; ^2^Shandong Provincial Key Laboratory of Endocrinology and Lipid Metabolism, Institute of Endocrinology and Metabolism, Shandong Academy of Clinical Medicine, Jinan, China; ^3^Shandong Clinical Medical Center of Endocrinology and Metabolism, Institute of Endocrinology and Metabolism, Shandong Academy of Clinical Medicine, Jinan, China; ^4^Department of Biostatistics, School of Public Health, Shandong University, Jinan, China; ^5^Department of Endocrinology, Shandong Provincial Hospital affiliated to Shandong First Medical University, Jinan, China; ^6^Department of Scientific Center, Shandong Provincial Hospital affiliated to Shandong First Medical University, Jinan, China

**Keywords:** statin, thyroid function, thyroid-stimulating hormone, total cholesterol, mediation analysis

## Abstract

**Purpose:**

Previous studies have suggested that cholesterol may influence thyroid function. Since statins are widely used for their cholesterol-lowering effect, we aimed to assess the association between statin use and thyroid function, and also to explore the role of the cholesterol-lowering effect in it.

**Methods:**

We performed a retrospective cohort study derived from REACTION study. Eligible subjects receiving statin therapy were included in the statin group, and sex-, age-, total cholesterol (TC)-, and thyroid function-matched participants without lipid-lowering therapy were included in the control group. The median follow-up time was three years. Outcomes of thyroid function were evaluated at the end of follow-up. We used multivariable regression models to assess the association between statin use and outcomes of thyroid function, and also performed mediation analyses to explore the role of cholesterol in it.

**Results:**

A total of 5,146 participants were screened, and 201 eligible subjects in the statin group and 201 well-matched subjects in the control group were analyzed. At the end of follow-up, TC and thyroid-stimulating hormone (TSH) levels in the statin group were lower than those in the control group (both p < 0.05), and the percentage of euthyroid subjects was higher in the statin group (88.06% vs. 76.12%, p = 0.002). The incidence rate of subclinical hypothyroidism (SCH) in euthyroid subjects was lower in the statin group (6.29% vs. 14.86%, p = 0.009), and the remission rate among subjects with SCH was higher in the statin group (50.00% vs. 15.38%, p = 0.008). In multivariable regression analyses, statin use was independently associated with lower TSH levels and higher odds to be euthyroid (OR 2.335, p = 0.004) at the end of follow-up. Mediation analyses showed the association between statin use and TSH levels were mediated by TC changes during follow-up.

**Conclusion:**

Statin use was associated with benefits of thyroid function, and TC changes serve as a mediator of the association between statin use and TSH levels. Further studies are needed to clarify the possible underlying mechanism.

## Introduction

Hypothyroidism is a common pathological condition of thyroid hormone deficiency, including overt hypothyroidism (OH) and subclinical hypothyroidism (SCH) (1). In China, the prevalence of SCH has significantly increased from 3.22% in 1999 to 16.7% in 2011 (2). The most frequent cause of SCH is Hashimoto’s thyroiditis in iodine-sufficient areas (3). However, risk factors contributing to the increasing prevalence of SCH remain unclear.

In recent years, studies have revealed the emerging role of the disturbance of lipid metabolism in the development of hypothyroidism (4–9). Our previous prospective observational study found that high baseline total cholesterol (TC) level was a risk factor of progression to OH in patients with SCH (10), which suggested that cholesterol may influence thyroid function. It is known that SCH is associated with an increased risk of cardiovascular disease (11, 12), and cholesterol is a key element in the development of cardiovascular disease (13). If cholesterol-lowering therapy can benefit thyroid function, we can not only find a possible way to relieve the disease burden of SCH, but also provide additional evidence that cardiovascular mortality and morbidity can be reduced by the proper control of cholesterol levels.

Statins are widely used due to their ability to lower cholesterol in clinical practice. Besides, statins also have pleiotropic actions such as anti-inflammatory and immunomodulatory properties (14, 15). Only a few studies have investigated the effects of statins on thyroid function, and the results were inconsistent (16–18). A reason for these findings could be due to small sample sizes that were limited to hospital-based patients only. It still remains inconclusive whether statin use is associated with improved thyroid function in the general population. We believe that it is worth clarifying this relationship, as well as investigating whether this is mediated by the cholesterol-lowering function of statins.

In this population-based retrospective cohort study, we aimed to assess the association between statin use and thyroid function, as well as to explore the role of the cholesterol-lowering effect in it.

## Materials and Methods

### Study Design and Participants

This study involves retrospective analyses of the population derived from the community-based REACTION study, which was a prospective observational cohort study in China investigating the epidemiology of metabolic diseases in residents aged 40 years or older (19). The study protocol was approved by the ethics committee of Shanghai Jiao Tong University, and all participants provided written informed consent before data collection.

In this study, data were obtained from participants who enrolled in REACTION study in Ningyang County, Shandong Province between April 2011 and July 2017. We included 5,146 participants who had more than one visit during the study period and assessed for eligibility. As our primary focus was the relationship between statin use and the outcome of thyroid function, we excluded subjects using the following exclusion criteria: (1) Missing vital data, such as age, sex, body mass index (BMI), or thyroid function; (2) self-report history of thyroid tumor, thyroidectomy, or radioactive iodine therapy; (3) intake of medications that influence thyroid function or serum lipids except statins (including thyroid hormone, antithyroid drugs, amiodarone, lithium, β-adrenergic blockers, fibrates, and steroid hormone) within the past 3 months; and (4) complications or conditions that affect thyroid status or lipid metabolism, such as pregnancy, lactation, severe liver dysfunctions (either alanine aminotransferase (ALT) or aspartate aminotransferase (AST) higher than 100 U/L), renal dysfunction (creatinine higher than 105 µmol/L and an estimated glomerular filtration rate (eGFR) generated from simplified MDRD equation below 60 ml/min), or malignant tumor (4).

**Figure 1:**
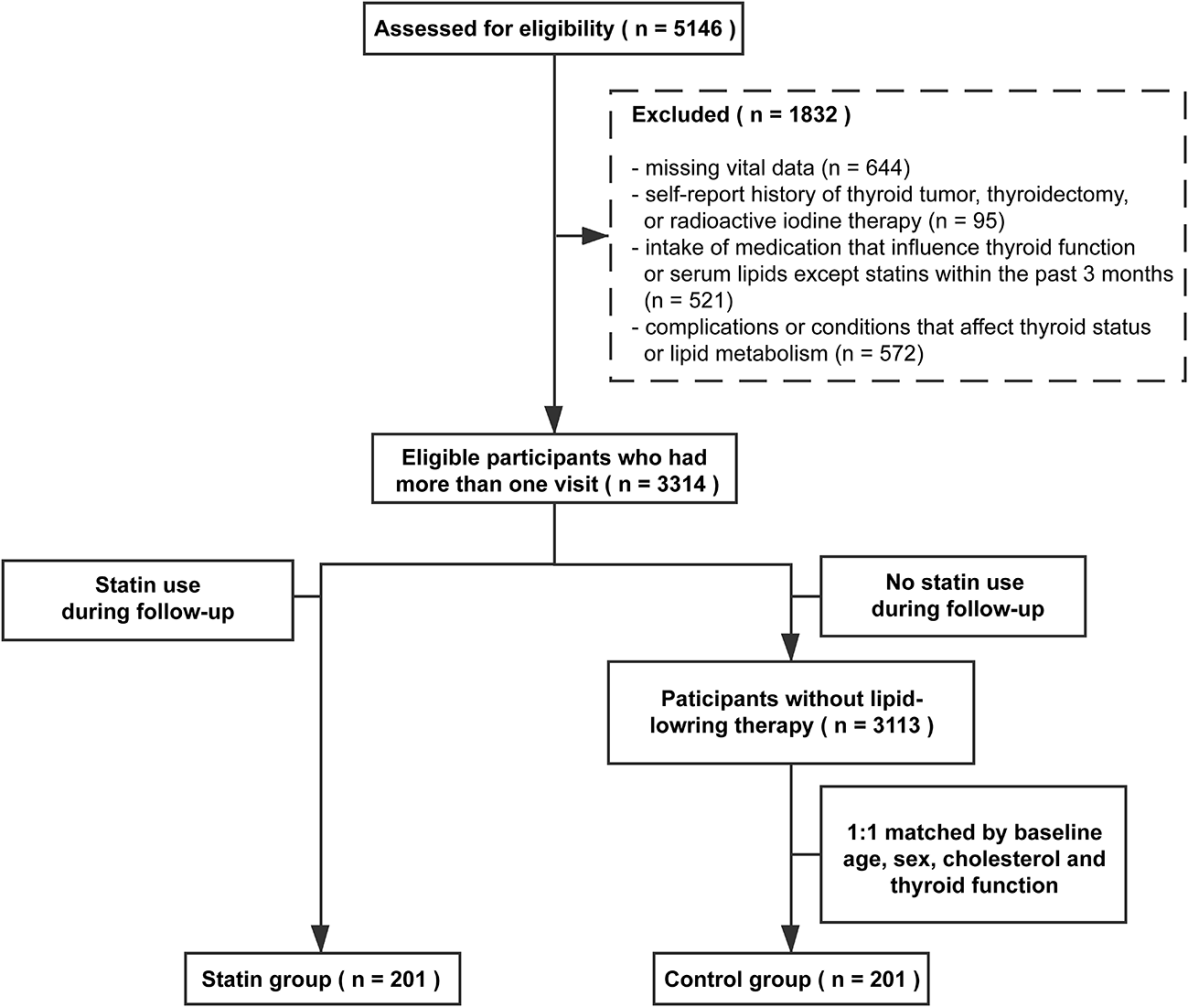
The flowchart of enrollment.

Participants who had statin therapy (including atorvastatin, fluvastatin, lovastatin, pitavastatin, pravastatin, rosuvastatin, and simvastatin) during the follow-up period were defined as the statin group. Considering non-random treatment allocation and potential confounding covariates, we used baseline sex-, age-, TC-, and thyroid function-matched participants without lipid-lowering therapy as the control group (1:1 match). Ultimately, 201 participants in the statin group and 201 participants in the control group were included in the final analysis. The selection process is illustrated in **Figure 1**.

### Data Collection

The data collection process has been described in the previous study (4). Briefly, all investigators went through a training program successfully to minimize instructor variability. Data collection was conducted at local health stations near the participants’ residential area. Trained investigators obtained information on demographic characteristics, medical history (including statin use), and other essential information from a well-established questionnaire through a face-to-face interview. Weight and height were measured in kilograms and centimeters, respectively. BMI was calculated by dividing weight by the square of the height. Blood pressure was measured three times using an electronic sphygmomanometer (HEM-7117; Omron, Kyoto, Japan) after 5 min rest, and the average of the three measurements was calculated. Above data were collected at baseline and each follow-up.

Blood samples were collected between 0800 h and 1000 h after at least 10-h fasting. Thyroid-stimulating hormone (TSH), free thyroxine (FT4), free triiodothyronine (FT3), antithyroglobulin antibody (TgAb), and thyroperoxidase antibody (TPOAb) were measured by chemiluminescence methods (Cobas E610, Roche, Basel, Switzerland). The serum lipid profile, hepatic function, and renal function were measured using the ARCHITECT ci16200 Integrated System (Abbott, Illinois, USA). Fasting plasma glucose (FPG) was measured within 2 h using the glucose oxidase method. The intra-assay and interassay coefficients of variation were always below 5% for all of the above parameters.

### Main Exposure and Study Outcomes

The exposure was statin use during follow-up. The primary outcome was thyroid status at the end of the follow-up. The laboratory reference ranges were 0.27–4.2 μIU/mL for TSH, 12–22 pmol/L for FT4, and 3.1–6.8 pmol/L for FT3. The reference range for TPOAb was 0–34 IU/L and for TgAb was 0–115 IU/L. Euthyroidism was defined as serum TSH, FT4, and FT3 levels within the reference ranges. SCH was defined as TSH >4.2 μIU/mL and FT4 levels within a reference range. OH was defined as TSH >4.2 μIU/mL and FT4 levels <12 pmol/L. The secondary outcome was serum TSH levels at the end of follow-up.

### Statistical Analysis

Continuous variables were expressed as means ± standard deviations (SD) or medians (interquartile ranges). Categorical variables were summarized as numbers (percentage). Differences of continuous variables between different groups were compared by using Student’s t-test or the Mann–Whitney test. For the comparison of measurements at baseline and follow-up, the Paired t-test and Wilcoxon matched-pair signed-rank test were employed. Differences of categorical variables were tested by using the chi-square test. We used logistic regression models to assess the relationship between statin use and thyroid status. We further used linear regression models to examine the relationship between statin use and log-transformed serum TSH levels at the end of follow-up. The potential confounders that may affect thyroid function were all adjusted in both the multivariate logistic regression models and the linear models, including age, sex, thyroid autoimmunity, BMI, SBP, FPG, ALT, and eGFR.

A mediation analysis was performed to examine whether the association of statin use with TSH levels was mediated by TC changes. **Supplementary Figure 1** shows the mediation model. A mediation effect was established when the model met Baron and Kenny’s principles (20): 1) the independent variable, statin use, significantly influenced the mediator, which is changes of TC in this model (path a); 2) the mediator significantly influenced the dependent variable, TSH levels at the end of follow-up (path b); 3) the independent variable significantly influenced the dependent variable when the mediator was not controlled (path c); 4) when path a and b were controlled, the significant relation was attenuated (partial mediation) or no longer significant (complete mediation) (path c’). Sobel test (21) was used to examine if the indirect effect was statistically significant.

A p-value < 0.05 was considered to indicate statistical significance, and all testing was two-sided. Confidence intervals (CIs) are reported as two-sided 95%. All statistical analyses were performed using SPSS version 24.0 for Windows.

## Results

**Figure 1** summarized the screening process of this study. A total of 5,146 participants were assessed for eligibility. Based on the self-reported medical history and biochemical measurements, 1832 participants were excluded according to our exclusion criteria. Among eligible participants, 201 of them used statins during the follow-up period and were identified as the statin group, including euthyroid subjects (n = 175) and subjects with SCH (n = 26) at baseline. The control group included 201 sex-, age-, TC- and thyroid function-matched subjects without lipid-lowering therapy. The median follow-up time of the study population was three years.

### Baseline Characteristics of Subjects in the Statin Group and the Control Group

**Table 1** presents the baseline characteristics of the participants in the statin group and the control group. Age, sex, TC, FT4, TSH, and thyroid antibodies were well matched between the two groups. Low-density lipoprotein cholesterol (LDL-C), FPG, ALT, AST, and follow-up time were comparable between the statin group and the control group. FT3, BMI, systolic blood pressure (SBP), and diastolic blood pressure (DBP) were lower in the control group, and eGFR was lower in the statin group (all *p* < 0.05). For the comorbidities, there are more people accompanied with cardiovascular disease (CVD), hypertension (HT), and diabetes mellitus (DM) in the statin group compared with the control group. Accordingly, the proportion of hypoglycemic medication use is also higher in the statin group (all *p* < 0.05). The characteristics of subjects in the statin group and the control group after follow-up were provided in **Supplementary Table 1**.

**Table 1:** Baseline characteristics of subjects in the statin group and the control group.

Characteristics	Statin Group (N = 201)	Control Group (N = 201)	*p* value^b^
Age (years), mean ± SD	57.36 ± 7.96	57.28 ± 7.95	0.925
Sex, n (%)			0.690
Male participants	97 (48.26%)	101 (50.25%)	
Female participants	104 (51.74%)	100 (49.75%)	
TC (mmol/L), mean ± SD	5.83 ± 1.55	5.73 ± 1.23	0.479
LDL-C (mmol/L), mean ± SD	3.48 ± 1.19	3.43 ± 0.99	0.645
FT_3_ (pmol/L), mean ± SD	4.94 ± 0.64	4.79 ± 0.58	0.016
FT_4_ (pmol/L), mean ± SD	16.76 ± 2.25	16.45 ± 2.04	0.151
TSH (μIU/mL), median (IQR)	2.14 (1.88)	2.17 (1.82)	0.939
Positive TgAb/TPOAb, n(%)^a^	16 (7.96%)	15 (7.46%)	0.852
BMI (kg/m^2^), mean ± SD	26.67 ± 3.34	24.95 ± 3.47	<0.001
SBP (mm Hg), mean ± SD	148.06 ± 20.61	140.58 ± 21.24	<0.001
DBP (mm Hg), mean ± SD	85.75 ± 12.21	82.18 ± 12.42	0.004
FPG (mmol/L), mean ± SD	6.93 ± 2.38	6.81 ± 2.33	0.617
ALT (IU/L), mean ± SD	19.90 ± 8.81	18.21 ± 8.83	0.056
AST (IU/L), mean ± SD	22.49 ± 8.01	22.63 ± 7.25	0.850
eGFR (ml/min/1.73m^2^), mean ± SD	101.61 ± 17.81	106.72 ± 18.66	0.005
CVD, n (%)	39 (19.40%)	8 (3.98%)	<0.001
HT, n (%)	97 (48.26%)	67 (33.33%)	0.002
DM, n (%)	93 (46.27%)	66 (32.84%)	0.006
Hypoglycemic medication, n (%)	34 (16.92%)	16 (7.96%)	0.007
Time (years), median (IQR)	2.71 (1.18)	2.78 (1.28)	0.069

TC, total cholesterol; LDL-C, low-density lipoprotein cholesterol; FT_3_, free triiodothyronine; FT_4_, free thyroxine; TSH, thyroid stimulating hormone; TgAb, antithyroglobulin antibody; TPOAb, thyroperoxidase antibody; BMI, body mass index; SBP, systolic blood pressure; DBP, diastolic blood pressure; FPG, fasting plasma glucose; ALT, alanine aminotransferase; AST, aspartate aminotransferase; eGFR, estimated glomerular filtration rate; CVD, cardiovascular disease; HT, hypertension; DM, diabetes mellitus; SD, standard deviation; IQR, interquartile range.

^a^Positive TgAb/TPOAb was defined as serum TPOAb/Tg Ab higher than their upper limits of reference ranges.

^b^Continuous variables were compared by using Student’s t-test or the Mann–Whitney test, and categorical variables by using the chi-square test. A p value < 0.05 were considered significant.

### Changes in TC Levels and Thyroid Function in the Statin Group and the Control Group

**Figure 2:**
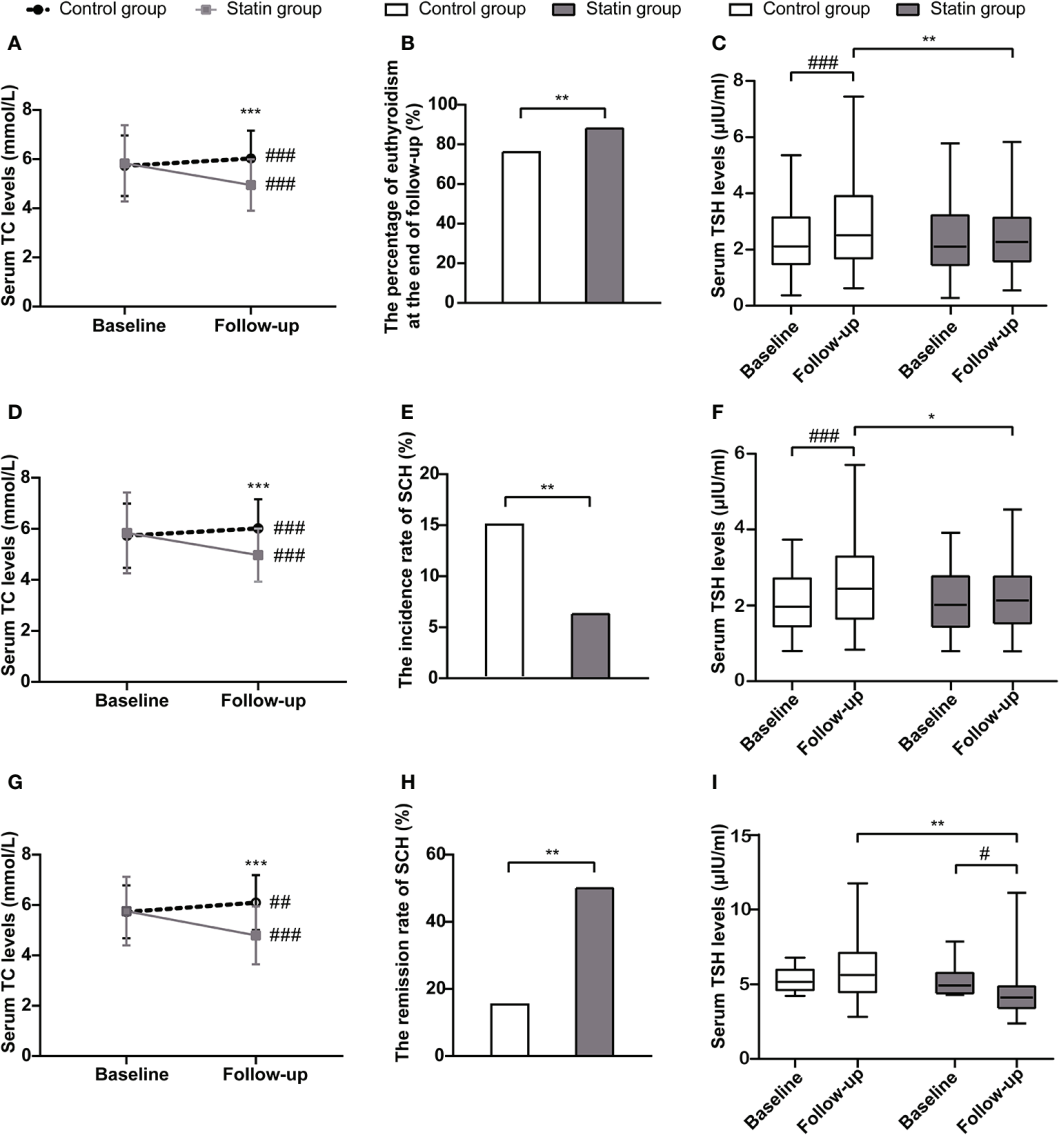
The changes of total cholesterol levels and thyroid function in the statin group and the control group in total population **(A–C)**, euthyroid subjects at baseline **(D–F)** and subjects with subclinical hypothyroidism (SCH) at baseline **(G–I)**. **(A)** Changes of serum total cholesterol levels from baseline to the end of follow-up in each group in total population. **(B)** The percentage of subjects with euthyroidism at the end of follow-up in each group in total population. **(C)** Changes of serum thyroid-stimulating hormone (TSH) levels from baseline to the end of follow-up in each group in total population. **(D)** Changes of serum total cholesterol levels from baseline to the end of follow-up in each group in euthyroid subjects at baseline. **(E)** The incidence rate of SCH during follow-up in each group in euthyroid subjects at baseline. **(F)** Changes of serum TSH levels from baseline to the end of follow-up in each group in euthyroid subjects at baseline. **(G)** Changes of serum total cholesterol levels from baseline to the end of follow-up in each group in subjects with SCH at baseline. **(H)** The remission rate of SCH during follow-up in each group in subjects with SCH at baseline. **(I)** Changes of serum TSH levels from baseline to the end of follow-up in each group in subjects with SCH at baseline. *p < 0.05, **p < 0.01, and ***p < 0.001 compared with the control group. ^#^*p* < 0.05, ^##^*p* < 0.01, and ^###^*p* < 0.001 compared with the baseline.

To evaluate the cholesterol-lowering effects of statin, we first analyzed the changes in serum TC levels during follow-up to see if there were any differences between the statin group and the control group. In the control group, serum TC levels increased from 5.73 ± 1.23 mmol/L to 6.03 ± 1.13 mmol/L (*p* < 0.001), while in the statin group, serum TC levels reduced from 5.83 ± 1.55 mmol/L to 4.95 ± 1.05 mmol/L (*p* < 0.001). At the end of follow-up, serum TC levels were significantly lower in the statin group than those in the control group (*p* < 0.001) (**Figure 2A****)**. At the same time, the percentage of normal thyroid function in the statin group was higher than that in the control group at the end of follow-up (88.06% *vs.* 76.12%, *p* = 0.002) (**Figure 2B**). As a more sensitive marker of thyroid dysfunction, the serum TSH levels in the control group elevated significantly during the follow-up period (*p* < 0.001) while remained steady in the statin group (*p* = 0.820) (**Figure 2C**). Accordingly, the serum TSH levels at the end of follow-up were significantly lower in the statin group compared with the control group (2.32 [1.72] μIU/mL vs. 2.61 [2.43] μIU/mL, *p* = 0.007) (**Figure 2C**).

We further did subgroup analyses according to the thyroid status of subjects at baseline. In both euthyroid subjects and subjects with SCH at baseline, serum TC levels were significantly lower in the statin group than those in the control group at the end of follow-up (**Figures 2D, G****)**. Among subjects with normal thyroid function at baseline, the incidence rate of SCH during follow-up was significantly lower in the statin group compared with the control group (6.29% *vs.* 14.86%, *p* = 0.009) (**Figure 2E**). Also, the TSH levels were significantly lower in the statin group than the control group at the end of follow-up (*p* < 0.001) (**Figure 2F**). Among subjects with SCH at baseline, the remission rate of SCH was 50% in the statin group during follow-up, while it was only 15.38% in the control group (*p* = 0.008) (**Figure 2H****)**. Consistently, the serum TSH levels in the statin group significantly reduced from 4.97 (1.54) μIU/mL to 4.19 (1.52) μIU/mL during follow-up, which did not have a significant change in the control group (5.2 [1.46] μIU/mL to 5.66 [2.95] μIU/mL) (**Figure 2I**). Besides, there were two subjects in the control group and one subject in the statin group developed OH during follow-up. Together, subjects in the statin group had better outcomes of thyroid function than the control group in both euthyroid and SCH subjects.

### Association Between Statin Use and the Outcomes of Thyroid Function

To evaluate the relationship between statin use and the outcomes of thyroid function and if it was independent of confounding factors, we utilized the logistic regression models. As presented in **Table 2**, subjects with statin use had higher odds of normal thyroid function at the end of follow-up compared with the control group (OR 2.314, 95%CI 1.354–3.953, *p* = 0.002). The results were similar after adjusting for age, sex, thyroid autoimmunity, BMI, SBP, FPG, ALT, and eGFR at baseline (OR 2.335, 95%CI 1.313–4.152, *p* = 0.004) and further adjustment for the presence of CVD and follow-up time (OR 2.510, 95%CI 1.380–4.564, *p* = 0.003) (**Table 2**).

**Table 2:** Logistic regression analysis of statin use and normal thyroid function at the end of follow-up.

	B	SE	OR	95% CI of OR	*p* value
Statin use in total population					
Univariable model	0.839	0.273	2.314	1.354–3.953	0.002
Multivariable model 1	0.848	0.294	2.335	1.313–4.152	0.004
Multivariable model 2	0.920	0.305	2.510	1.380–4.564	0.003
Statin use in euthyroid subjects					
Univariable model	0.956	0.377	2.602	1.242–5.448	0.011
Multivariable model 1	0.882	0.394	2.416	1.116–5.230	0.025
Multivariable model 2	0.949	0.410	2.582	1.156–5.770	0.021
Statins use in subjects with SCH					
Univariable model	1.705	0.670	5.500	1.478–20.461	0.011
Multivariable model 1	1.955	0.964	7.066	1.068–46.758	0.043
Multivariable model 2	2.177	1.059	8.824	1.106–70.377	0.040

Dependent variable: normal thyroid function at the end of follow-up; 1 = maintenance of normal thyroid function in euthyroid subjects; 1 = normalization of thyroid function in subjects with SCH.

Data are coefficient (B), corresponding standard error (SE), odds ratio (OR), 95% confidence interval (CI) of OR, and significance (p value).

Multivariable model 1 was adjusted for basal age, sex, thyroid autoimmunity, body mass index, systolic blood pressure, fasting plasma glucose, alanine aminotransferase, and estimated glomerular filtration rate;

Multivariable model 2 was further adjusted for the presence of cardiovascular disease and follow-up time.

We also performed logistic regression analysis with normal thyroid function as dependent variable and statin use as independent variable in euthyroid subjects and subjects with SCH at baseline, respectively. We found that statin use was significantly associated with normal thyroid function at the end of follow-up in both subgroups (**Table 2**). After adjusting for confounding factors, the association remained significant. Compared with individuals without statin use, the odds of maintenance of normal thyroid function for statin use increased 2.416-fold in euthyroid subjects, and the odds of normalization of thyroid function increased 7.066-fold for statin use in subjects with SCH at baseline after adjustment for potential confounders (**Table 2**). The results remained similar after further adjustment for the presence of CVD and follow-up time. These results suggested that statin use was independently associated with a better chance of normal thyroid function at the end of follow-up in both SCH and euthyroid participants at baseline.

### Linear Regression Analysis of Statin Use and Serum TSH Levels at the End of Follow-Up

As TSH is a more sensitive marker of thyroid dysfunction, we further performed linear regression analysis to examine the relationship between statin use and serum TSH levels at the end of follow-up. We found that statin use was negatively associated with log-transformed TSH levels at the end of follow-up (*p* < 0.006). After adjustment for age, sex, thyroid hormone, TSH, thyroid autoimmunity, BMI, SBP, FPG, ALT, and eGFR at baseline, statin use was independently and negatively associated with the log-transformed TSH levels at the end of follow-up (*p* = 0.002) (**Table 3**). The results remained similar after further adjustment for the presence of CVD and follow-up time (**Table 3****)**.

**Table 3:** Linear regression analysis of statin use and log-transformed thyroid-stimulating hormone (TSH) levels at the end of follow-up.

	B	SE	95% CI of B	Beta	*p* value
Statin use in total population					
Univariable model	−0.073	0.027	−0.125 to −0.021	−0.136	0.006
Multivariable model 1	−0.061	0.020	−0.100 to −0.022	−0.114	0.002
Multivariable model 2	−0.053	0.020	−0.093 to −0.013	−0.099	0.010
Statin use in euthyroid subjects					
Univariable model	−0.068	0.026	−0.119 to −0.017	−0.139	0.009
Multivariable model 1	−0.052	0.021	−0.093 to −0.011	−0.106	0.014
Multivariable model 2	−0.045	0.021	−0.087 to −0.003	−0.092	0.035
Statin use in subjects with SCH					
Univariable model	−0.108	0.053	−0.214 to −0.001	−0.276	0.047
Multivariable model 1	−0.131	0.066	−0.265 to 0.002	−0.337	0.053
Multivariable model 2	−0.116	0.071	−0.261 to 0.028	−0.298	0.111

TSH, thyroid stimulating hormone.

Data are unstandardized coefficients (B), corresponding standard error (SE), 95% confidence interval (CI) of B, standardized coefficients (Beta), and significance (p value).

Multivariable model 1 was adjusted for basal age, sex, free triiodothyronine, free thyroxine, thyroid stimulating hormone, thyroid autoimmunity, body mass index, systolic blood pressure, fasting plasma glucose, alanine aminotransferase, and estimated glomerular filtration rate;

Multivariable model 2 was further adjusted for the presence of cardiovascular disease and follow-up time.

Similar results are achieved in the subgroup analysis of euthyroid subjects at baseline. In the multivariable models, statin use was negatively associated with the log-transformed TSH level at the end of follow-up (*p* = 0.014 in model 1 and *p* = 0.035 in model 2) (**Table 3**). In subjects with SCH at baseline, statin use also suggested the trend of a negative association with serum log-transformed TSH levels at the end of follow-up (**Table 3****)**. These results demonstrated that statin use was associated with a decrease in TSH levels, even in euthyroid subjects.

### Mediation Analysis of TC Changes in the Relationship Between Statin Use and TSH Levels at the End of Follow-Up

**Figure 3:**
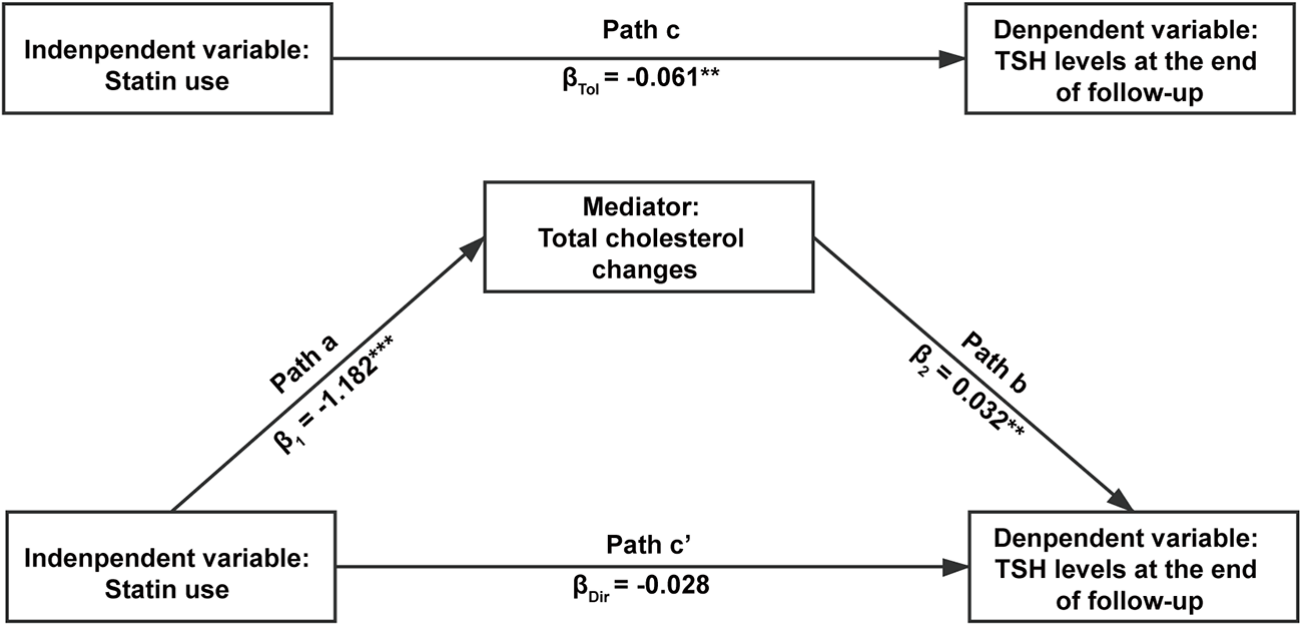
The mediation effect of total cholesterol changes in the relationship between statin use and log-transformed thyroid-stimulating hormone (TSH) levels at the end of follow-up. The independent variable was statin use, the dependent variable was serum TSH levels at the end of follow-up, and the mediator was the changes of serum total cholesterol levels during follow-up. Age, sex, free triiodothyronine, free thyroxine, thyroid stimulating hormone, thyroid autoimmunity, body mass index, systolic blood pressure, fasting plasma glucose, alanine aminotransferase and evaluated glomerular filtration rate at baseline were adjusted in the analysis. In path a, the mediator was regressed onto the independent variable. In path b, the dependent variable was regressed onto the mediator. In path c, the dependent variable was regressed onto the independent variable without the adjustment of mediator. In path c’, the dependent variable was regressed onto the independent variable with the adjustment of mediator. **p < 0.01 and ***p < 0.001.

Since previous studies suggested that cholesterol may influence thyroid function and we already found an association between statin use and benefits of thyroid function, we performed a mediation analysis to test if TC changes mediated the relationship between statin use and TSH levels at the end of follow-up. In the crude model, the total effect of statin use on TSH levels is significant (*p* = 0.006), and mediation analysis showed the effect was indirect and completely mediated by TC changes (*p* = 0.024) (**Table 4**). After adjustment for age, sex, thyroid hormone, TSH, thyroid autoimmunity, BMI, SBP, FPG, ALT, and eGFR at baseline, the results were similar (**Table 4**, **Figure 3**): the total effect of statin use on log-transformed TSH levels at the end of follow-up is significant (path c, β_Tol_ = -0.061, *p* = 0.002). Statin use also significantly associated with TC changes (path a, β_1_ = -1.182, *p* < 0.001), and TC changes significantly associated with log-transformed TSH levels at the end of follow-up (path b, β_2_ = 0.032, *p* = 0.001). After adjustment for TC changes, the direct effect of statin use on log-transformed TSH levels at the end of follow-up was no longer significant (path c’, *p* = 0.201). The result of Sobel test suggested a significant indirect effect (β_Ind_ = −0.038, *p* = 0.001). According to Baron and Kenny’s principals, these results indicated a complete mediation effect of TC changes in the relationship between statin use and TSH levels at the end of follow-up. A similar mediation effect was also found in euthyroid subjects at baseline (**Supplementary Figure 2** and **Supplementary Table 2**).

**Table 4:** Mediation analysis of total cholesterol changes in the relationship between statin use and log-transformed thyroid-stimulating hormone (TSH) levels at the end of follow-up.

	B	SE	*p* value
Crude model^a^			
Total effect	−0.073	0.027	0.006
Direct effect	−0.038	0.031	0.214
Indirect effect	−0.035	0.016	0.024
Multivariable model^b^			
Total effect	−0.061	0.020	0.002
Direct effect	−0.028	0.022	0.201
Indirect effect	−0.038	0.012	0.001

TSH, thyroid stimulating hormone.

Data are coefficients (B), corresponding standard error (SE), and significance (p value).

^a^Dependent variable: TSH levels at the end of follow-up; independent variable: statin use; mediating variable: changes of total cholesterol.

^b^Multivariable model was adjusted for basal age, sex, free triiodothyronine, free thyroxine, thyroid-stimulating hormone, thyroid autoimmunity, body mass index, systolic blood pressure, fasting plasma glucose, alanine aminotransferase, and estimated glomerular filtration rate.

## Discussion

In our present study, we investigated whether statin use is associated with a beneficial effect on thyroid function in a community-based population. We found that the percentage of euthyroid subjects was higher in the statin group at the end of follow-up. In multivariable regression analyses, statin use was independently associated with normal thyroid function and lower TSH levels at the end of follow-up. Furthermore, the mediation analysis showed that the relationship between statin use and lower TSH levels was mediated by TC declines. Collectively, our results suggest that statin use is indeed associated with benefits of thyroid function, and TC changes serve as a mediator.

The prevalence of SCH has significantly increased in China in the past decades (2), which suggests the importance of identifying the potential risk factors. At the same time, dyslipidemia is also a concerning problem in the world (22). Both SCH and hypercholesteremia increase the risk of cardiovascular events (3, 22), and these two conditions can present simultaneously in many cases (3). Evidence from animal study has suggested that hypercholesteremia could influence thyroid function (23), and our previous population study also found that higher TC level was an independent risk factor of progression to OH in SCH patients (10). If the control of TC levels could also benefit thyroid function, this could be important to clinical practice and may be valuable to our management strategy of hypothyroidism. On the basis of previous studies, we found that the cholesterol-lowering effect of statin was associated with better outcomes of thyroid function in both SCH and euthyroid subjects. Our findings provide additional evidence for the hypothesis that the disturbance of lipid metabolism may cause thyroid dysfunction, and stress the importance of controlling TC levels. SCH (including mild SCH) was related to a higher risk of cardiovascular events and mortality, even after adjusting for other cardiovascular risk factors including LDL-C (12). LDL-C is a known independent risk factor for cardiovascular diseases. If LDL-C lowering agents, like statins, can not only lower LDL-C levels, but also maintain normal thyroid function, this study may provide additional evidence for the use of statins to prevent cardiovascular disease. Additionally, it may provide novel mechanisms that statins lowering the risk of cardiovascular disease, which are through the management of thyroid function.

To our knowledge, this is the first study exploring the relationship between statin use and thyroid function in a community-based population and analyzing the role of cholesterol-lowering effects played in it. So far, the few studies that have investigated the relationship between statin therapy and thyroid function are only focused on patients with Hashimoto’s thyroiditis. A clinical trial by Sevim Gullu, et al. included 21 patients with SCH aged between 28 and 48 years, and they also found an improvement in the thyroid function after Simvastatin treatment for eight weeks (16). However, Robert Krysiak, et al. did not found significant changes in thyroid function after the treatment of statins (17, 18). The impact of these studies was limited by their small sample size and study population, which was only patients with Hashimoto’s thyroiditis but not the general population. Different from these previous hospital-based studies, our study is a community-based retrospective study in the general population. Taking that hypothyroidism is a chronic disease, the absolute change of TSH levels during follow-up was modest since nearly 90% of our study subjects were euthyroid at baseline. However, the percentage of normal thyroid function in the statin group was significantly higher than that in the control group at the end of follow-up, indicating that the change of TSH was clinically significant. Our results showed that statin use was independently associated with normalization of thyroid function in subjects with SCH at baseline, which were similar to Sevim Gullu’s study. Also, in subjects who were euthyroid at baseline, we found that statin use was independently associated with the maintenance of euthyroidism, which represents a lower risk of progression to SCH.

As statins are known to have not only a cholesterol-lowering effect but also pleiotropic actions, we used mediation analysis to further explore if the cholesterol-lowering effect mediated the association between statin use and thyroid function in our study. Since serum TSH level is a more sensitive marker of thyroid state, we used TSH levels at the end of follow-up as the dependent variable. The results of the mediation analysis indicated a complete mediation effect of TC changes in the relationship between statin use and TSH levels at the end of follow-up. In our subgroup analysis of euthyroid subjects at baseline, TC changes still serve as a mediator of the association between statin use and TSH levels at the end of follow-up. In one study from Robert Krysiak, et al., which included euthyroid women with plasma lipids within the reference range, there were no differences found in TSH and free thyroid hormones between atorvastatin-treated women and atorvastatin-naïve women (18). This result does not conflict with our findings, and it implies that cholesterol changes may be necessary for the relationship between statin use and thyroid function, as the plasma lipids in their participants remained at similar levels during the study period. In the subgroup of subjects with SCH at baseline, we did not perform mediation analysis because the statin use did not significantly associate with low TSH levels at the end of follow-up when the mediator was not controlled yet (*p* = 0.053). However, the lack of statistical significance in this subgroup analysis should be taken with caution, as this is likely due to the insufficient sample size of subjects with SCH at baseline in our study. Besides, we also compared the changes of thyroid autoantibodies in the statin group and the control group after follow-up. Although in the statin group TgAb titer reduced after follow-up, there are no significant changes in the positivity rates for TgAb/TPOAb during follow-up in both the statin group and the control group (data not shown). Collectively, these suggest TC changes serve as a mediator of the association between statin use and TSH levels.

Increasing evidence has shown that the disturbance of lipid metabolism might play a role in the pathogenesis of thyroid dysfunction (5–8, 24). As one kind of lipid components, cholesterol might also affect thyroid function, just like saturated fats. Ayuob et al. have reported the negative impact of high cholesterol diet on thyroid function and structural changes in rats, which could be ameliorated by the administration of grape juice accompanied with amelioration of hypercholesterolemia (23). However, the underlying mechanism is still not clear. Previous studies found that excess cellular cholesterol could induce endoplasmic reticulum (ER) stress (25, 26), which is related to the development of hypothyroidism (24, 27). A possible explanation is that ER stress can down-regulate the expression of key genes involved in thyroid hormone synthesis in FRTL-5 thyrocytes (28). In our animal study, we found that LDL receptor and Niemann-Pick C1-like1 (NPC1L1), both of which are crucial cholesterol receptors, were expressed in the thyroid glands of SD rats, and high cholesterol diet could influence thyroid function by inducing ER stress (data not shown). Thus, ER stress could be one potential mechanism between cholesterol and thyroid function. Other possible mechanisms involved in this pathological process might include mitochondrial oxidative stress, and so on. Further investigations are essential to clarify the effect and potential mechanisms of cholesterol and cholesterol-lowering effects on thyroid function.

Our study has several strengths. First, this is the first study exploring the relationship between statin use and thyroid function in a community-based cohort. Second, our study innovatively found that statin use is associated with benefits of thyroid function and TC changes serve as a mediator. Finally, our finding has potential meaning to clinical practice and may be valuable to our management strategy of hypothyroidism. Still, our study has some limitations which need to be pointed out. First, it is important to note that due to the nature of an observational study, causal relationships cannot be established. Although we have used baseline matched control and multivariable model to adjust for the known confounding factors, we cannot exclude the unknown confounding factors and potential reverse causation. Randomized controlled trials and basic research are needed to further clarify the effect of statin on thyroid function and the underlying mechanisms. Also, since this is not a randomized controlled trial and creatine kinase was not measured, we do not suggest using statin monotherapy for patients with hypothyroidism to correct their thyroid function before more evidence on its safety and efficacy has been obtained. Second, the definition of hypothyroidism is based on a single thyroid function test, without a second confirmatory finding of elevated TSH levels. As the serum TSH concentration can be transiently elevated, some of our participants may have been misclassified. Although the intra-assay and inter-assay coefficients of variation were below 5% for all parameters in our study, and our blood samples were collected between 8 a.m. and 10 a.m. after at least 10-h fasting, we should also keep in mind that assay variation or diurnal variation may influence the results to some extent. Last, although our study population was derived from a large community-based study, the sample size of our study population is still relatively small because statin use in the community-based population is limited. Besides, as detailed information on statin use was not available in this retrospective cohort study, further study is needed to give a better insight into the relationship between statin use and thyroid function.

In conclusion, this retrospective study demonstrated that statin use was independently associated with better outcomes of thyroid function, and TC changes serve as a mediator of the association between statin use and TSH levels at the end of follow-up. The obtained results provide some evidence that controlling serum TC levels may benefit thyroid function in the general population, which may have implications for the role of cholesterol in the etiology of SCH and put more emphasis on the importance of cholesterol-lowering therapy in subjects with a high risk of developing SCH. More studies are needed to further clarify the effect of cholesterol-lowering therapy on thyroid function and the underlying mechanisms.

## Data Availability Statement

The data sets presented in this article are not readily available because the ethical approval obtained for this study prevents the human data being shared publicly to protect patients’ privacy. Requests to access the data sets would be passed to the ethics committee who will decide whether they can access the data directly. Requests to access the data sets should be directed to JZ, jjzhao@sdu.edu.cn.

## Ethics Statement

The studies involving human participants were reviewed and approved by the ethics committee of Shanghai Jiao-Tong University. The patients/participants provided their written informed consent to participate in this study.

## Author Contributions

JZ, MZ, and LG contributed to conception and design of the study. YWa, SM, SS, YWu, ZW, and QiuL organized the database. YWa, QiL, ZY, and MZ performed the statistical analysis. YWa wrote the first draft of the manuscript. QiL, JZ, MZ, and LG contributed to manuscript revision. All authors contributed to the article and approved the submitted version.

## Funding

This research was supported by grants from the National Key Research and Development Program of China (2017YFC1309800) and the National Natural Science Foundation (81430020, 81922016, 81900793, 81600604, and 91957209).

## Conflict of Interest

The authors declare that the research was conducted in the absence of any commercial or financial relationships that could be construed as a potential conflict of interest.
