# Supplementary material for: Statin Use and Benefits of Thyroid Function: A Retrospective Cohort Study
Source: Front Endocrinol (Lausanne). 2021 Mar 2;12:578909. doi: 10.3389/fendo.2021.578909 (PMC7962670; doi:10.3389/fendo.2021.578909)
Supplement: Supplementary file 1 [file DataSheet_1.docx]

Supplementary Material

# 1 Supplementary Tables

**Supplementary Table 1.** Characteristics of subjects in the statin group and the control group at the end of follow-up

| Characteristics | Statin Group  (N=201) | Control Group  (N=201) | *p* value |
| --- | --- | --- | --- |
| TC (mmol/L), mean ± SD | 4.95±1.05 | 6.03±1.13 | <0.001 |
| LDL-C (mmol/L), mean ± SD | 2.76±0.84 | 3.57±0.92 | <0.001 |
| FT_3_ (pmol/L), mean ± SD | 5.13±2.48 | 4.95±0.49 | 0.310 |
| FT_4_ (pmol/L), mean ± SD | 16.63±6.36 | 16.35±2.21 | 0.547 |
| TSH (IU/ml), median (IQR) | 2.32 (1.72) | 2.61 (2.43) | 0.007 |
| BMI (kg/m^2^), mean ± SD | 26.81±3.61 | 25.67±4.57 | 0.006 |
| SBP (mmHg), mean ± SD | 139.47±22.10 | 136.67±20.07 | 0.235 |
| DBP (mmHg), mean ± SD | 81.28±11.23 | 79.01±11.58 | 0.072 |
| FPG (mmol/L), mean ± SD | 6.88±2.19 | 6.94±2.83 | 0.809 |
| ALT (IU/L), mean ± SD | 21.49±9.58 | 19.96±11.74 | 0.156 |
| AST (IU/L), mean ± SD | 23.18±7.90 | 23.51±8.54 | 0.694 |
| eGFR (mL/min/1.73m^2^), mean ± SD | 96.14±18.37 | 98.50±16.86 | 0.182 |

Abbreviations: TC, total cholesterol; LDL-C, low-density lipoprotein cholesterol; FT_3_, free triiodothyronine; FT_4_, free thyroxine; TSH, thyroid stimulating hormone; BMI, body mass index; SBP, systolic blood pressure; DBP, diastolic blood pressure; FPG, fasting plasma glucose; ALT, alanine aminotransferase; AST, aspartate aminotransferase; eGFR, estimated glomerular filtration rate; CVD, cardiovascular disease; HT, hypertension; DM, diabetes mellitus; SD, standard deviation; IQR, interquartile range.

**Supplementary Table 2.** Mediation analysis of total cholesterol changes in the relationship between statin use and log-transformed TSH levels at the end of follow-up in euthyroid subjects at baseline.

|  | B | SE | *p* value |
| --- | --- | --- | --- |
| Crude model^a^ |  |  |  |
| Total effect | -0.068 | 0.026 | 0.009 |
| Direct effect | -0.035 | 0.029 | 0.228 |
| Indirect effect | -0.032 | 0.014 | 0.026 |
| Multivariable model^b^ |  |  |  |
| Total effect | -0.052 | 0.021 | 0.014 |
| Direct effect | -0.023 | 0.023 | 0.315 |
| Indirect effect | -0.035 | 0.012 | 0.004 |

Abbreviations: TSH, thyroid stimulating hormone.

Data are coefficients (B), corresponding standard error (SE) and significance (P value).

^a^Dependent variable: TSH levels at the end of follow-up; independent variable: statin use; mediating variable: changes of total cholesterol.

^b^Multivariable model was adjusted for basal age, sex, free triiodothyronine, free thyroxine, thyroid stimulating hormone, thyroid autoimmunity, body mass index, systolic blood pressure, fasting plasma glucose, alanine aminotransferase and estimated glomerular filtration rate.

## 2 Supplementary Figures


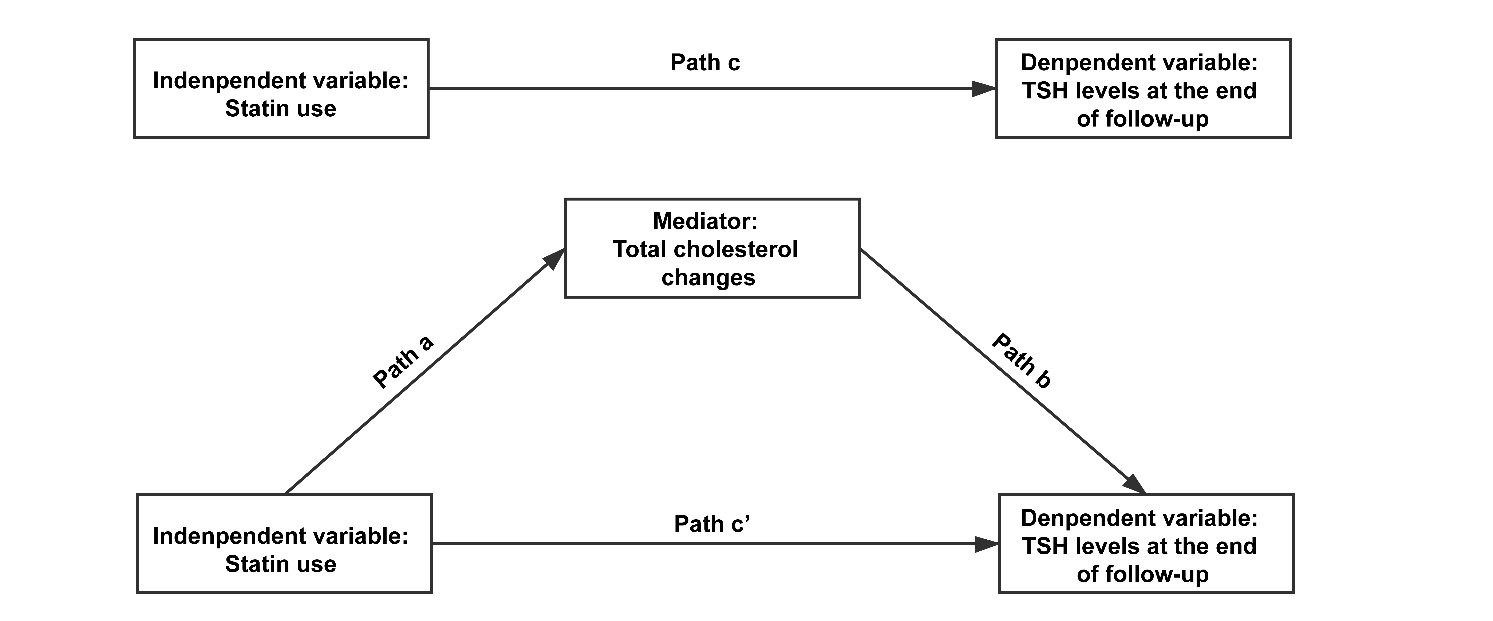


**Supplementary Figure 1.** The mediation model illustrating relationship between statin use, changes of serum total cholesterol levels and serum TSH levels at the end of follow-up.


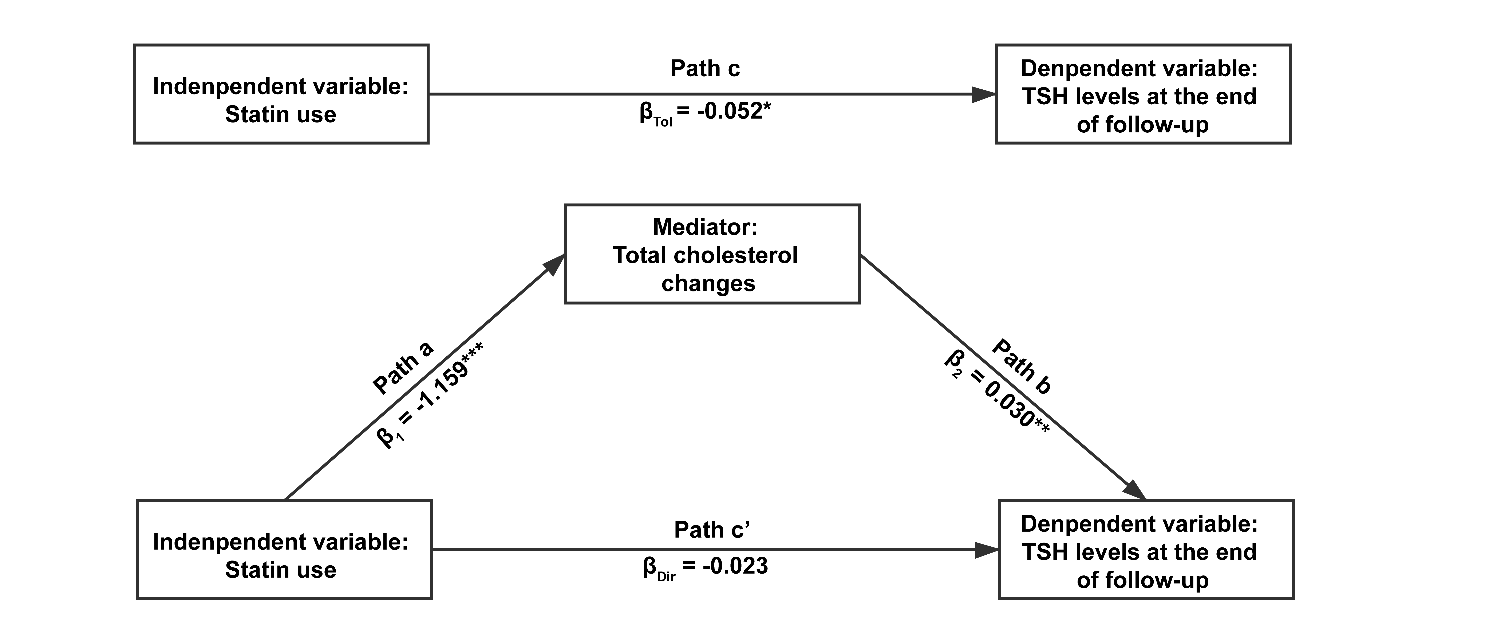


**Supplementary Figure 2.** The mediation effect of serum total cholesterol in the relationship between statin use and TSH levels at the end of follow-up in the subgroup of enthyroid subjects. The independent variable was statin use, the dependent variable was serum TSH levels at the end of follow-up, and the mediator was the changes of serum total cholesterol levels during follow-up. Age, sex, free triiodothyronine, free thyroxine, thyroid stimulating hormone, thyroid autoimmunity, body mass index, systolic blood pressure, fasting plasma glucose, alanine aminotransferase and evaluated glomerular filtration rate at baseline were adjusted in the analysis. In path a, the mediator was regressed onto the independent variable. In path b, the dependent variable was regressed onto the mediator. In path c, the dependent variable was regressed onto the independent variable without the adjustment of mediator. In path c’, the dependent variable was regressed onto the independent variable with the adjustment of mediator. **p* <0.05, ***p* < 0.01 and ****p* < 0.001.
